# A pilot study of safety of sulfamethoxazole, trimethoprim and guaifenesin in pediatric and adult patients with acute bronchitis

**DOI:** 10.1186/s13104-019-4150-2

**Published:** 2019-03-04

**Authors:** Mayer Falcon, Carlos Iberico, Frances Guerra, Isabel Reyes, Efraín Felix, Mónica Flores, Jorge de los Ríos, Maria E. Diaz, Alberto Casas, Sergio Sanchez-Gambetta, Roberto Carrasco

**Affiliations:** 1 Clínica Internacional, Av. Guardia Civil #385, San Borja, Lima 41, Peru; 2 Clínica Providencia, Calle Carlos Gonzalez # 250, San Miguel, Lima 32, Peru; 3 Clínica Anglo-Americana, Calle Alfredo Salazar #350, San Isidro, Lima 27, Peru; 4 Hospital de Emergencias Pediátricas, Prolongación Huamanga #138, La Victoria, Lima 13, Peru; 5 Clínica San Borja, Av. Guardia Civil 337, Lima 41, Peru; 6 Clínica de Especialidades Médicas, Jr.Eduardo Ordoñez #468, Lima 41, Peru; 7 Hospital de Apoyo María Auxiliadora, Av. Miguel Iglesias #968, Lima 29, Peru; 8 Roche–Peru, Av Dionisio Derteano #144, San Isidro, Lima 27, Peru

**Keywords:** Acute bronchitis, Sulfamethoxazole, Trimethoprim, Guaifenesin, Safety, Adverse events

## Abstract

**Objective:**

This exploratory study assessed the safety of the combination of sulfamethoxazole, trimethoprim and guaifenesin (STG) in adult and pediatric patients with acute bronchitis according to local labelling in Peru.

**Results:**

We enrolled 51 pediatric and 52 adult participants diagnosed with acute bronchitis and indication of STG. The mean ages were 7.6 years (SD ± 3.2 years) and 42.8 years (SD ± 16.1) and the proportion of female patients were 51% and 65%, respectively. The duration of treatment in pediatric patients was < 5 days in 2% of patients, 5 days in 13.7%, 6–7 days, in 82.4% and > 7 days in 2% while in adults patients it was < 5 days in 17%, 5 days in 69.2%; 6–7 days in 28.8% of patients. Adverse events (AEs) were registered in 9.6% and 19.2% of pediatric and adult patients, respectively. These AEs had definite relation of causality with the study drugs in 2 adults (20% of AEs) and possible causality with the study drugs in 4 pediatric (80% of AEs) and 2 adult cases (20% of AEs). Our results provide valuable data to develop trials of pharmacovigilance where different statistical parameters should be considered to calculate an adequate sample size in studies evaluating STG in pediatric or adult patients.

*Trial registration* NCT02879981 and NCT02902640

**Electronic supplementary material:**

The online version of this article (10.1186/s13104-019-4150-2) contains supplementary material, which is available to authorized users.

## Introduction

Acute bronchitis is a respiratory tract infection limited to large airways of the lung in the absence of chronic lung disease affecting people of all ages and it is one of the most frequent medical conditions seen in ambulatory care. Clinically, acute bronchitis is characterized by cough with or without sputum production lasting 1 to 3 weeks [1–3].

The two most important meta-analyses that evaluated the use of antibiotics in acute bronchitis have not proven a clear benefit of these drugs; however, a weakness of these studies was the pooled of trials evaluating different type or combination of antibiotics. Despite it, a modest benefit in the use of antibiotics in certain subgroups of patients was described [4, 5].

Regardless of the current trend to decrease the use of antibiotics in this pathology, the combination of sulfamethoxazole, trimethoprim and guaifenesin (STG) is frequently used in the routine practice in Peru to treat acute respiratory tract infections, including acute bronchitis, similarly to other countries where antibiotics are used into treat these conditions [6–8].

Although the STG combination is widespread used in the routine, there is a lack of data about the safety of this combination. For this reason, we designed an exploratory trial to estimate a frequency of adverse events (AEs) associated with the treatment with STG that in order to estimate of a robust sample size for future studies evaluating the safety of this combination.

## Main text

### Methods

#### Study design

This is a prospective, observational, multicenter, pilot study in pediatric and adult patients with acute bronchitis intended to explore the safety of the STG combination.

#### Patients

This study included patients with acute bronchitis in which the treating physician has decided to initiate treatment with the combination of STG. We describe in this report results of two cohorts of patients (adult and pediatric). Eligibility criteria for both cohorts are described in Additional file 1: Table S1.

#### Treatment schedule

Administration of STG was done following physicians’ recommendation according to local clinical practice and local protocols.

#### Evaluations

The security assessment criteria were registered. The primary variable was number of adverse events related to the STG combination (as assessed by the investigator according to the NCI-CTCAE). Study variables also included, change in dosage, change in frequency, clinical parameters affecting the safety of the combination, discontinuation of the dose, dose reintroduction, and compliance with local recommendations. The safety assessment criteria were evaluated in the final visit.

#### Statistical considerations

We present descriptive statistics, mean and standard deviation (SD) for quantitative data and frequencies for qualitative variables. This study planned evaluate variables associated to AEs; however, due to few number of events with definite/possible causality, it analysis was not conducted.

The calculation of the sample size was made in the following formula

$$ {\rm N} = \ln ( 1- \pi) / \ln ( 1- \lambda ) $$

where ln = natural logarithm, π = confidence level and λ = probability of event.

To make the calculation, the following considerations were proposed: π = 0.95 (95%) and λ = 0.06 (6%). The result of the calculation is 48.4 patients. The premise of a probability adverse-drug reaction of 6% was obtained in a previous article by Nguyen et al. [9], describing an adverse drug reaction rate of 9% in a cohort of patients treated with high and standard doses of the combination of sulfamethoxazole and trimethoprim where the frequency of adverse reactions for standard dose was 5.1%.

### Results

#### Characteristic of patients

In total, 51 pediatric patients and 52 adult patients were included in every cohort, where 49% (n = 25) and 61.5% (n = 32) were female patients. The mean age was 7.6 years (SD ± 3.2) in pediatric patients and 42.8 years (SD ± 16.1) in adult patients. In regard to the body mass index (BMI), 39.2% of pediatric patients and 59.5% were over weighted/obese. Symptoms observed with more frequency in the pediatric cohort were cough (n = 51, 100%), fever (n = 32, 63%) and coryza (n = 51%); while most frequent symptoms in the adult cohort were cough (n = 51, 98.1%), dyspnea (n = 32, 61.5%), fever (n = 29, 55.8%), coryza and dysphonia (both in 48.1%) (Table 1).

**Table 1:** Baseline characteristics of both pediatric and adult patients

Characteristics	Pediatric patients		Adult patients	
	n	%	n	%
*Gender*				
Female	25	49	32	61.5
Male	26	51	20	38.5
*Age*				
Mean (years) ± SD	7.6 ± 3.2		42.8 ± 16.1	
*Weight*				
Mean (years) ± SD	29.9 ± 13		68.9 ± 14.4	
*Height*				
Mean (years) ± SD	1.3 ± 0.17		1.6 ± 0.08	
*BMI*				
Low weight	1	2	1	1.9
Normal	30	58.8	20	38.5
Overweight	9	17.6	21	40.4
Obesity	11	21.6	10	19.1
*Symptoms*				
Cough	51	100	51	98.1
Fever	32	63	29	55.8
Coryza	26	51	25	48.1
Dysphonia	9	18	25	48.1
Dyspnea	8	16	32	61.5
Chest wall retraction	2	4	2	3.8
Other symptoms	27	53	16	30.8

#### Features of treatment

In pediatric patients, duration of treatment was < 5 days in one case, 5 days in 13.7% (n = 7); between 6 and 7 days, in 82.4% (n = 42) and > 7 days in one case. In the adult group of patients, duration of treatment was < 5 days in 32.7% of patients (n = 17), 5 days in 69.2% (n = 27) and between 6 and 7 days in 28.8% (N = 8) (Table 2). At the last follow-up visit, 98% of pediatric patients (n = 50) and 98.1% of adult patients (n = 51) completed their treatment at their last follow-up. In total, 80.4% (n = 41) and 65.4% (38%) of pediatric and adult patients received concomitant medications, respectively. In all cases treatment was carried out under local labeling.

**Table 2:** Treatment characteristics of both cohorts

Characteristics	Pediatric patients		Adult patients	
	n	%	n	%
*Treatment days*				
< 5	1	2	17	32.7
5	7	13.7	27	69.2
6–7	42	82.4	8	28.8
> 7	1	2	0	_
*Completed treatment* ^a^				
Yes	50	98	51	98.1
No	1	2	1	1.9
*Interruption of treatment*				
Yes	1	2	2	3.8
Reason				
Adverse event	1	100	1	50
Improvement of condition	_		1	50
*Concomitant medication*				
Yes	41	80.4	38	65.4
No	10	19.6	18	34.6

^a^Treatment completed at the last follow up (day 10th)

#### Adverse events

AEs were present in 9.6% (n = 5) of pediatric and 19.2% (n = 10) of adult patients. Regarding the causality, AEs assigned as definite causality were present in 2 adult patients (gastritis and gastric discomfort); possible, in 4 pediatric (diarrhea) and 2 adult patients (nausea and vomiting and pyrosis); probable, in adult patients (headache, diarrhea, gastritis and nocturia); unlikely, in 1 pediatric (prurigo) and 2 adult patients (xerostomy and insomnia). AEs features are presented in Table 3. Interruption of treatment due to AEs was observed in one pediatric and one adult patient. In none case was observed change of dose, change of frequency or treatment reintroduction.

**Table 3:** Adverse events in both pediatric and adult patients

Characteristics	Pediatric patients		Adult patients	
	n	%	n	%
*Adverse events*				
Yes	5	9.6	10	19.2
No	46	88.4	42	80.8
*Causality*				
Definite	0	_	2	20
Possible	4	80	2	20
Probable	0	_	4	40
Unlikely	1	20	2	20
None	0	_	0	_
*Outcome of adverse event*				
Total recovery	4	80	7	70
Recovering^a^	1	20	2	20
Recovery with sequelae	0	_	0	_
Without recovery^a^	0	_	1	10
Fatal	0	_	0	_

^a^Assessed at the last day of follow-up (day 10th)

### Discussion

Although, the most commonly etiology of acute bronchitis is viral infections, a community study conducted by Macfarlane et al. [10], found 25% of cases with a bacterial infection cause, with *Streptococcus pneumoniae*, *Haemophilus influenza*, *Moraxella catarrhalis*, as the most frequent pathogens. In this, the combination of sulfamethoxazole and trimethoprim is used in the clinical setting to cover the infections [11]. In the other hand, guaifenesin has a well-established and favorable safety and tolerability profile in adult and pediatric patients and could improve treatment outcomes adding symptoms relief in the management of chronic bronchitis and upper respiratory tract infections and in patients-reported outcomes for acute respiratory tract infection symptoms [12, 13].

The use of antibiotics to treat acute bronchitis is controversial but common. In a recent study in Italy, 73.5% of events of acute bronchitis had antibiotic treatment where fluoroquinolones were the most used antibiotics by general practitioners [14]. In regard to pediatric patients, the analysis of a large cohort involving 14,683 episodes of acute bronchitis, described that antibiotics were prescribed in 49.7% of cases [15].

In this study of observational design in the context of routine setting in seven healthcare centers in Peru, we evaluated the safety of the combination of STG in both, pediatric and adult patients. In this work, we present the first report of the safety of the combination of sulfamethoxazole, trimethoprim and guaifenesin.

Up to date, there is scarce information in the medical literature about the therapeutic combination evaluated in this study while systematic reviews and meta-analysis evaluated pool of antibiotics rather specific interventions [1]. The first Cochrane meta-analysis evaluating antibiotics in acute bronchitis were published in 2014, describing modest benefit to some group of patients [4]. An update of this meta-analysis published 3 years later, do not included additional studies and had similar conclusions than the previous report [4].

In our study, the pattern of treatment vary between adult and pediatric patients, where adults had longer treatments and where less likely to receive concomitant medication, although a similar rate of patients with completed treatment were observed.

On the other hand, we found the double rate of adverse events in adults in contrast to pediatric patients (≈ 10% vs ≈ 20%); however, it could be explained for a higher description of adverse events less likely to be related with the study drugs. Diarrhea was most frequent in pediatric patients while adult patients had a higher frequency of gastritis.

In this exploratory study, rates of adverse event was higher than reported previously to the combination of sulfamethoxazole and trimethoprim [9]; however, these results should be taken carefully interpreted because this work is only designed to obtain a sample size for a further study.

In conclusion, the combination of STG has a good toxicity profile we report that different statistical considerations should be taken to calculate adequate sample sizes to evaluate the safety of the combination of sulfamethoxazole plus trimethoprim plus guaifenesin in adults or pediatric patients.

## Limitations

- This study had a small sample size to draw conclusions about associations between basal characteristics and the likelihood to develop AEs.
- The follow-up period was short (10 days) and it difficult the monitoring of adverse events.

## Additional file

**Additional file 1: Table S1.** Eligibility criteria. Description of inclusion and exclusion criteria in paediatric and adult patients enrolled in this study.
