# Supplementary material for: A pilot study of safety of sulfamethoxazole, trimethoprim and guaifenesin in pediatric and adult patients with acute bronchitis
Source: BMC Res Notes. 2019 Mar 4;12:119. doi: 10.1186/s13104-019-4150-2 (PMC6399863; doi:10.1186/s13104-019-4150-2)
Supplement: Supplementary file 1 — Additional file 1: Table S1. Eligibility criteria. Description of inclusion and exclusion criteria in paediatric and adult patients enrolled in this study. [file 13104_2019_4150_MOESM1_ESM.docx]

**Table S1.- Eligibility criteria**

| COHORT | INCLUSION CRITERIA | EXCLUSION CRITERIA |
| --- | --- | --- |
| Pediatric patients | Patients aged 4 to 14 years old | Patients who have started treatment with another antibiotic in the query time. |
|  | Patients initiating treatment with the combination sulfamethoxazole, trimethoprim,guaifenesin | Patients without respiratory infections. |
|  | Patients with a clinical diagnosis of acute bronchitis (cough with or without sputum less than 30 days) according to medical judgment. | Patients who do not accept entering the study. |
|  |  | Patients who do not accept to enter the study or the legal guardian reject the participation of the patient |
|  |  | Patients with severe hepatic parenchymal damage. |
|  | Legal guardian and the patient (if age appropriate) understand the nature of the study and are willing to meet evaluations associated monitoring, and provide written informed consent and assent before the procedure) | Patients with severe renal insufficiency if the plasma concentration of drugs cannot be monitored. |
|  |  | Patients with hypersensitivity to any of its active ingredients, excipients and / or sulfa drugs |
| Adult patients | Patients aged ≥ 18 years old | Patients who have started treatment with another antibiotic in the query time |
|  | Patients initiating treatment with sulfamethoxazole, trimethoprim/ guaifenesin | Patients without respiratory infections. |
|  | Patients with a clinical diagnosis of acute bronchitis (cough with or without sputum less than 30 days) according to medical judgment. | Patients who do not accept entering the study. |
|  |  | Patients with mental disorders, who do not allow the clinical evaluation of the patient according to the criteria of the attending physician. |
|  |  | Patients with severe hepatic parenchymal damage. |
|  | Patients who have received oral and written information about the study and are not opposed to allowing their health information to be processed automatically (that is, they have given their informed consent). | Patients with severe renal insufficiency if the plasma concentration of drugs cannot be monitored |
|  |  | Patients with hypersensitivity to any of its active ingredients, excipients and / or sulfa drugs |
